# A novel application of PageRank and user preference algorithms for assessing the relative performance of track athletes in competition

**DOI:** 10.1371/journal.pone.0178458

**Published:** 2017-06-02

**Authors:** Clive B. Beggs, Simon J. Shepherd, Stacey Emmonds, Ben Jones

**Affiliations:** 1 Institute for Sport, Physical Activity and Leisure, Carnegie Faculty, Leeds Beckett University, Leeds, West Yorkshire, United Kingdom; 2 Medical Biophysics Laboratory, University of Bradford, Bradford, United Kingdom; East China University of Science and Technology, CHINA

## Abstract

Ranking enables coaches, sporting authorities, and pundits to determine the relative performance of individual athletes and teams in comparison to their peers. While ranking is relatively straightforward in sports that employ traditional leagues, it is more difficult in sports where competition is fragmented (e.g. athletics, boxing, etc.), with not all competitors competing against each other. In such situations, complex points systems are often employed to rank athletes. However, these systems have the inherent weakness that they frequently rely on subjective assessments in order to gauge the calibre of the competitors involved. Here we show how two Internet derived algorithms, the PageRank (PR) and user preference (UP) algorithms, when utilised with a simple ‘who beat who’ matrix, can be used to accurately rank track athletes, avoiding the need for subjective assessment. We applied the PR and UP algorithms to the 2015 IAAF Diamond League men’s 100m competition and compared their performance with the Keener, Colley and Massey ranking algorithms. The top five places computed by the PR and UP algorithms, and the Diamond League ‘2016’ points system were all identical, with the Kendall’s tau distance between the PR standings and ‘2016’ points system standings being just 15, indicating that only 5.9% of pairs differed in their order between these two lists. By comparison, the UP and ‘2016’ standings displayed a less strong relationship, with a tau distance of 95, indicating that 37.6% of the pairs differed in their order. When compared with the standings produced using the Keener, Colley and Massey algorithms, the PR standings appeared to be closest to the Keener standings (tau distance = 67, 26.5% pair order disagreement), whereas the UP standings were more similar to the Colley and Massey standings, with the tau distances between these ranking lists being only 48 (19.0% pair order disagreement) and 59 (23.3% pair order disagreement) respectively. In particular, the UP algorithm ranked ‘one-off’ victors more highly than the PR algorithm, suggesting that the UP algorithm captures alternative characteristics to the PR algorithm, which may more suitable for predicting future performance in say knockout tournaments, rather than for use in competitions such as the Diamond League. As such, these Internet derived algorithms appear to have considerable potential for objectively assessing the relative performance of track athletes, without the need for complicated points equivalence tables. Importantly, because both algorithms utilise a ‘who beat who’ model, they automatically adjust for the strength of the competition, thus avoiding the need for subjective decision making.

## Introduction

Ranking is an important task that enables coaches, applied scientists, sporting authorities, and pundits to determine the relative performance of individual athletes and teams in comparison to their peers or competitors. Ranking within talent identification, can also provide an objective assessment of the performance of young athletes relative to their peers, something that can be helpful when making important decisions about career progression [1, 2]. While ranking is a relatively straightforward task in sports that employ traditional leagues in which all the teams play each other (e.g. soccer and rugby), it is more difficult in sports that involve knock-out tournaments (e.g. tennis, international soccer) or are event based (e.g. athletics). With these, not all the athletes or teams play each other and as a result competition can become fragmented, making it difficult to assess performance relative to competitors. The situation is compounded by the fact that in many sports (e.g. athletics, tennis, golf) not all the athletes compete in every competition. Consequently, we can have the paradoxical situation where an athlete may appear to be performing well, having achieved several wins against low ranking opposition, while a much better athlete, who has entered just a few competitions, is ranked far below them despite having only narrowly lost to opponents of the highest calibre. As such, this may lead to a ‘false positive’ identification (i.e. the identification of an athlete who is not as good as their ranking suggests) or a ‘false negative’ (i.e. failure to identify an athlete who may be better than their ranking suggests), both situations that are undesirable. In situations where assessing the relative performance of competitors is difficult, developing a robust ranking system that accurately reflects the true performance of the respective athletes or teams represents a considerable mathematical challenge. If a ranking system is too simplistic, then it will fail to capture the complexities of the system and will struggle to reflect the true performance of the athletes concerned, something that may cause it to become discredited. Aware of this, many sporting authorities employ complex points based systems [3], which attempt to mirror the complexities associated with the competition structure. While these systems aim to be objective, they inevitably involve a degree of subjectivity when it comes to allocating the number of points to particular tournaments, with the result that the overall ranking process can be somewhat arbitrary. However, in recent years advances in computer science have yielded techniques, such as the Google PageRank (PR) algorithm, that have the potential to overcome this problem and make ranking a more objective process.

Accurate ranking of teams and athletes is something that poses a considerable challenge for many sporting authorities, and there is no clear consensus regarding the best approach that should be taken. In most sports, ranking is based on the accumulation of points awarded for performance during matches or tournaments. Leagues, such as those found in soccer and rugby, represent a classic example of this, with all the teams playing each other over the course of a season and the number of points accumulated indicating the respective standings. However, even within a league system such as this, the points system used can still vary between sports. For example: rugby league award two points for a win, one point for a draw and zero points for a loss [4]; soccer award three points for a win, one point for a draw and zero points for a loss [5]; while rugby union award two points for a win, one point for a draw and zero points for a loss, with the addition of a ‘bonus’ point system [6]. Golf and tennis, although not league based, also employ a points system. However, unlike the relatively simple points system employed in the leagues associated with the aforementioned sports, golf and tennis employ complex models which calculate the number of points accumulated in a rolling period. In golf [7] and tennis [8] the points awarded for the various competitions will tend to vary depending on the prestige of the event and the perceived strength of the competitors involved. Prestigious international tournaments will naturally yield more points in comparison to smaller local events. In athletics the International Association of Athletics Federations (IAAF) also employs a complex points system, which takes into account the measured result of athletes and their placings, together with the prestige and quality of the event in which they are competing. The process is complex and relies on published tables (e.g. Ref: IAAF Scoring Tables of Athletics [3]) to compute the respective points scored. While these point based systems are generally able to differentiate high performers from those who are more mediocre, they tend to be over-complex, difficult to understand, and can be somewhat arbitrary in their points allocation. As such, they are a relatively ‘blunt instrument’ and are therefore of only limited value to coaches seeking to assess the true performance of both developing and high-performance athletes.

In recent years graph theory has yielded a number of algorithms that have proved remarkably successful in computer science. Perhaps the most pre-eminent of which is the PR algorithm used to power the Google search engine. This algorithm, first developed at Stanford University by Larry Page and Sergey Brin in 1996 [9, 10], enables Google’s search engine to measure of the relative importance of every web page on the Internet. It does this by computing an adjacency matrix, which it then uses to determine the PR of each individual web page. The PR of any particular web page is calculated based on the quantity and PR quality of the incoming links to it. For any given page, the higher the PR of the incoming links, and the fewer outbound links associated with the page, the higher the PR given to the web page. Such is the robustness and power of the algorithm, that, for any given query, Google is able to rapidly rank web sites in order of importance, despite the huge complexity of the Internet (i.e. many millions of web pages). As such, the PR algorithm appears to have great potential as a tool for ranking athletes and sporting teams. In particular, because the algorithm can automatically evaluate relationships between athletes/teams irrespective of the quality of the tournament, it has the potential to remove much of the arbitrary decision-making currently associated with many ranking methodologies. However despite its potential, surprisingly few studies have investigated the use of the PR algorithm in a sporting context. For example, Zack et al [11] and Govan et al [12, 13] both used the PR algorithm to rank the performance of teams in the National Football League (NFL) in the USA, while Lazova and Basnarkov [14] used it to rank international soccer teams using Federation International Football Association (FIFA) World Cup data. Pena and Touchette [15] and Brandt and Brefeld [16] also used the PR algorithm, but did so to rank the performance of individual soccer players. Others have adapted the PR algorithm in an attempt to utilize it for prediction purposes [17]. However these studies have focused almost exclusively on sports in which games consists of two teams playing each other, with the result that the potential for the PR algorithm in athletics has been overlooked. Unlike team sports where each game involves only two teams, athletic events involve many individuals competing at the same time, making assessment more challenging for some ranking algorithms. However, this is not a problem for the PR algorithm, which was specifically designed to assess complex networks involving numerous interactions. We therefore designed the study presented here, with the specific aim of using the PR algorithm to evaluate the relative performance of male 100m sprinters throughout the course of the 2015 IAAF Diamond League season. These races were selected for investigation because: (i) they represented a closed system with well-defined outcomes; and (ii) the competition exhibited considerable asymmetry, with some athletes (e.g. Mike Rogers, Nesta Carter) competing many times, while others (e.g. Marvin Bracy, Usain Bolt) ran only once. As such, the system posed considerable challenges from a ranking standpoint, making it an ideal context with which to evaluate the PR algorithm. In addition, we adapted an Internet retail user preference (UP) algorithm [18], which we also used to rank the athletes. UP algorithms have some attributes that are well suited to sporting events where many individuals compete at the same time, and so we were interested to know if they could be adapted to successfully rank track athletes.

In order to assess the performance of the Internet derived algorithms, we also used three well-known sports ranking systems, the Colley [19], Massey [20] and Keener [21] algorithms, which we used for comparative purposes. These algorithms were originally developed for use in team sports where matches consist of two teams playing each other. We therefore had to adapt these algorithms so that they could be applied to track athletics.

## Method

The paper reports on a project that involved analysis of publicly available secondary data. Ethical approval for the project was granted by the Research Ethics Board of Leeds Beckett University.

### A. Data

Public domain data from IAAF Diamond League website [22] were collated for the ten Diamond League events (i.e. Doha, Eugene, Rome, Birmingham, New York, Paris, Lausanne, Monaco, London and Bruxelles) that included a male 100m race during the 2015 season. For each event, only the results of the A race were utilised, with the times and placings compiled into a single dataset (Table 1). The data were evaluated using firstly the PR algorithm, and then a UP algorithm, developed for Internet shopping [18], which we adapted to evaluate the track events. We also used adapted versions of the Colley, Massey and Keener algorithms to assess the data. All the algorithms were executed using bespoke ‘in-house’ programs written in Matlab (version R2016b; Mathworks, Natick, USA) and ‘R’ (version 3.3.3; open source statistical software).

**Table 1:** The results of the ten male 100m races listed on the IAAF Diamond League web site.

	Doha	Eugene*	Rome	Birmingham	New York	Paris	Lausanne*	Monaco	London*	Bruxelles
	15^th^ May	30^th^ May	4^th^ June	7^th^ June	13^th^ June	4^th^ July	9^th^ July	17^th^ July	24^th^ July	11^th^ Sept
Name	Position [Time (s)]	Position [Time (s)]	Position [Time (s)]	Position [Time (s)]	Position [Time (s)]	Position [Time (s)]	Position [Time (s)]	Position [Time (s)]	Position [Time (s)]	Position [Time (s)]
Harry Adams	na	na	9 [10.24]	na	na	na	na	na	na	na
Nickel Ashmeade	na	na	na	na	6 [10.28]	na	na	6 [10.11]	na	na
Guy-Elphege Anouman	na	na	na	na	na	9 [10.32]	na	na	na	na
Kemar Bailey-Cole	na	na	na	na	na	na	na	na	3 [9.92]	na
Deondre Batson	7 [10.10]	na	6 [10.08]	na	5 [10.24]	na	na	na	na	na
Emmanuel Biron	na	na	na	na	na	8 [10.18]	na	7 [10.17]	na	na
Keston Bledman	3 [10.01]	na	na	na	2 [10.13]	na	5 [10.03]	5 [10.10]	na	na
Usain Bolt	na	na	na	na	na	na	na	na	1 [9.87]	na
Marvin Bracy	na	na	na	1 [9.93]	na	na	na	na	na	na
Nesta Carter	6 [10.07]	5 [10.02]	4 [10.06]	4 [10.00]	3 [10.15]	4 [10.02]	na	na	7 [10.08]	na
Kim Collins	4 [10.03]	4 [9.99]	5 [10.07]	na	na	5 [10.05]	6 [10.08]	na	8 [10.09]	na
James Dasaolu	8 [10.14]	6 [10.13]	na	na	na	na	na	na	9 [10.19]	na
Andrew Fisher	na	na	8 [10.14]	na	na	na	na	na	na	na
Julian Forte	na	na	na	7 [10.15]	na	na	na	na	na	na
Justin Gatlin	1[9.74]	na	1 [9.75]	na	na	na	1 [9.75]	1 [9.78]	na	1 [9.98]
Tyson Gay	na	1 [9.88]	na	na	1 [10.12]	na	3 [9.92]	2 [9.97]	na	na
Adam Gemili	na	na	na	2 [9.97]	na	na	na	na	na	na
Ramon Gittens	na	na	na	na	na	na	na	na	na	6 [10.11]
Richard Kilty	na	na	na	5 [10.05]	na	na	na	na	na	na
Trell Kimmons	na	na	na	na	7 [10.40]	na	na	na	6 [10.07]	na
Churandy Martina	na	na	na	na	na	7 [10.12]	na	na	na	na
Joseph Morris	na	na	na	na	8 [10.45]	na	na	na	na	na
Femi Ogunode	5 [10.04]	na	na	na	na	na	na	na	na	2 [9.98]
Asafa Powell	na	na	na	na	na	1 [9.81]	2 [9.92]	na	na	5 [10.04]
Mike Rodgers	2 [9.96]	2 [9.90]	3 [9.98]	3 [9.97]	na	3 [9.99]	4 [10.03]	na	2 [9.90]	4 [10.02]
Akani Simbine	na	na	6 [10.08]	na	4 [10.18]	na	na	na	na	7 [10.18]
Bingtian Su	na	3 [9.99]	na	na	na	na	na	na	na	na
Richard Thompson	na	7 [10.27]	na	na	na	na	na	na	na	na
Chijindu Ujah	na	na	na	6 [10.11]	na	na	na	4 [10.08]	4 [9.96]	8 [10.19]
Clayton Vaughn	na	na	na	na	na	6 [10.08]	na	na	5 [9.98]	na
Jimmy Vicaut	na	na	2 [9.98]	na	na	2 [9.86]	na	3 [10.03]	na	3 [9.99]
Justin Walker	na	8 [10.28]	na	na	na	na	na	na	na	na
Isiah Young	na	na	na	na	na	na	7 [10.11]	na	na	na

* Races not included in the official 2015 IAAF Diamond League standings.

### B. PageRank algorithm

The PR algorithm was developed by Larry Page and Sergey Brin, the founders of the company Google, to rank web pages on the Internet [9]. The algorithm utilises graph theory, in so much that it relies on a directed adjacency matrix, *A*, which describes the relationships between web pages. In the graph described by the adjacency matrix, the web pages are nodes and the links between the web pages are the directed edges. The number of links leaving from any given node is called the ‘out-degree’ of that node [12]. These can be viewed as ‘votes’ cast by that web page in favour of other web pages. With the PR algorithm, the rank of any given web page is dependent on how many other pages ‘vote’ for it (i.e. are linked to it), and the rank of these other pages. A web page is considered important if it is pointed to by other web pages of importance.

With highly inter-connected webs such as the Internet, there is a high degree of recursiveness, with web pages often linked to each other in both directions, so that rank of one page often depends on the rank of the other and vice versa. To solve this type of system, an iterative procedure is required to determine the rank of all the pages in the web. In this process the PR of a web page, *P*, after k+1 iterations is:

$$r_{k+1}(P)=\sum_{Q\in B_{p}}\frac{r(Q)}{\left|Q\right|}$$ (1)

where, *r(P)* is the rank of the web page, *P*; *B*_*p*_ is the set of all the web pages pointing to *P*; and *|Q|* is the out-degree of a web page *Q* [12]. In order to complete the iterative procedure it is necessary to initialise the system by setting the initial ranks of *P* and *Q* to 1/n, where *n* is the number of web pages in the network.

**Fig 1:**
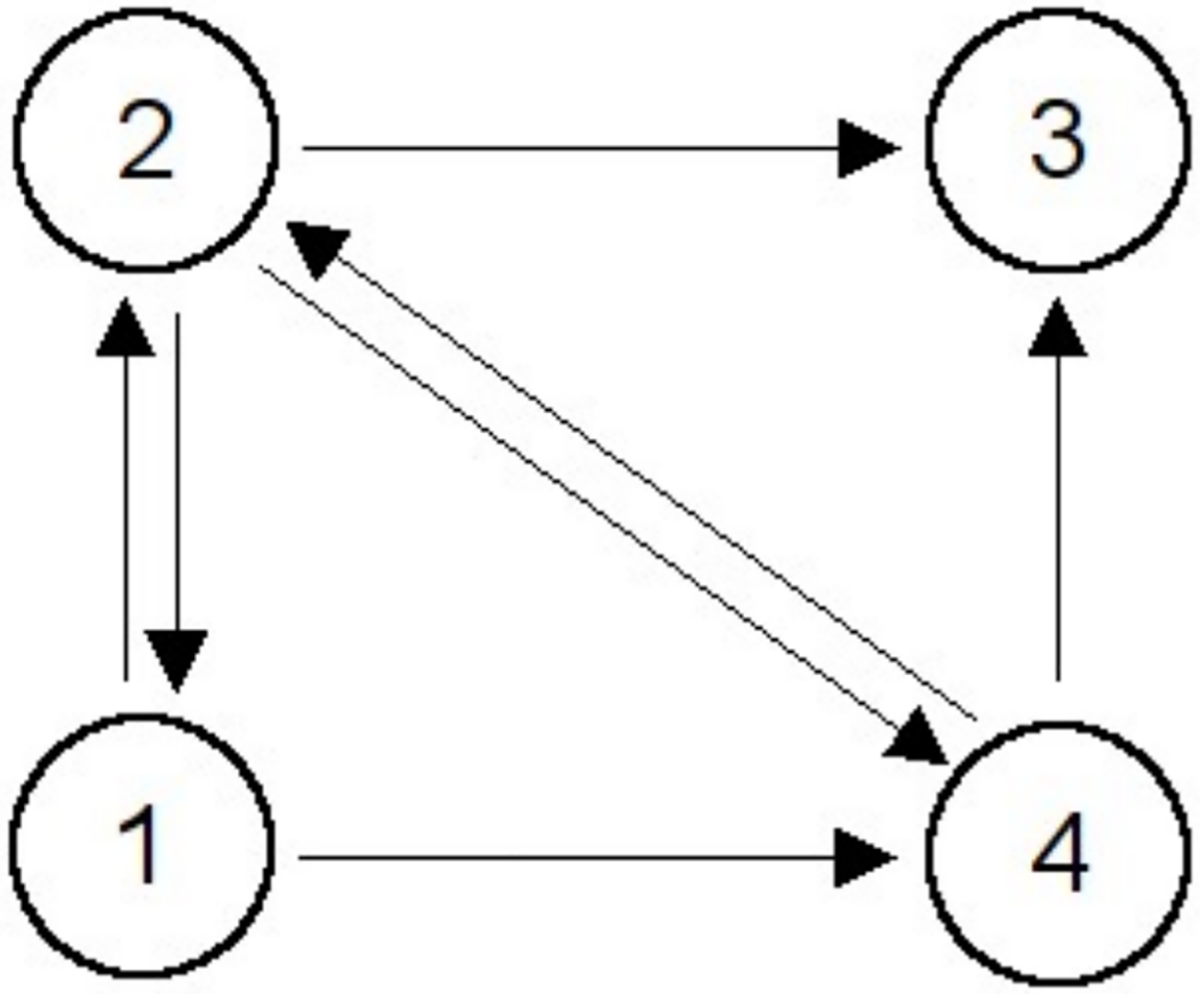
Small web containing just four web pages.

In order to explain the matrix algebra required to solve the PR problem, we consider the small web shown in Fig 1. In the adjacency matrix, *A*, summarising the graph structure of this web, if an edge (i.e. link) exists from node *P*_*i*_ to node *P*_*j*_, then 1 is inserted in the matrix, otherwise 0 is inserted. The resulting adjacency matrix, *A*, is:

$$A=\left[\begin{array}{cccc} 0 & 1 & 0 & 1\\ 1 & 0 & 1 & 1\\ 0 & 0 & 0 & 0\\ 0 & 1 & 1 & 0 \end{array}\right]$$ (2)

Matrix *A* is then divided by the number of out-degrees per page to produce the hyperlink matrix, *H*.

$$H=\left[\begin{array}{cccc} 0 & \frac{1}{2} & 0 & \frac{1}{2}\\ \frac{1}{3} & 0 & \frac{1}{3} & \frac{1}{3}\\ 0 & 0 & 0 & 0\\ 0 & \frac{1}{2} & \frac{1}{2} & 0 \end{array}\right]$$ (3)

Because the PR model requires a stochastic matrix in which all the row sums are equal to 1, it is necessary to adjust any rows containing all zeros (i.e. rows relating to nodes with no out-degrees). This is done by replacing any zero rows in H with 1/n to produce the stochastic matrix, *S*.

$$S=\left[\begin{array}{cccc} 0 & \frac{1}{2} & 0 & \frac{1}{2}\\ \frac{1}{3} & 0 & \frac{1}{3} & \frac{1}{3}\\ \frac{1}{4} & \frac{1}{4} & \frac{1}{4} & \frac{1}{4}\\ 0 & \frac{1}{2} & \frac{1}{2} & 0 \end{array}\right]$$ (4)

Finally, in order to ensure that the graph is strongly inter-connected and irreducible, the stochastic matrix, *S*, is modified using Eq (5) to produce the Google matrix, *G*.

G=αS+(1−α)E (5)

Where, α is a damping factor, usually set at 0.85; and *E* is a [n×n] matrix populated entirely with the value 1/n.

$$G=\left[\begin{array}{cccc} 0.0375 & 0.4625 & 0.0375 & 0.4625\\ 0.3208 & 0.0375 & 0.3208 & 0.3208\\ 0.2500 & 0.2500 & 0.2500 & 0.2500\\ 0.0375 & 0.4625 & 0.4625 & 0.0375 \end{array}\right]$$ (6)

The vector containing the final PR scores, *q*, is then computed using the power method shown in Eq (7).

$$q=z_{0}G^{k}$$ (7)

Where, *q* is a [1×n] vector containing the PR scores; *z*_*0*_ is a [1×n] vector containing the initial estimated PR scores, generally populated entirely with the value 1/n; and *k* is the number of iterations necessary to reach convergence.

For the small web in Fig 1, after convergence the PR vector, q, is:

$$q=\left[\begin{array}{cccc} 0.1782 & 0.2819 & 0.2861 & 0.2539 \end{array}\right]$$ (8)

From q it can be seen that node 3 has the highest PR, because it had no out-degrees, whereas node 1 was ranked lowest, reflecting the fact that it has 50% more links leaving the node than entering it.

### C. Application of the PageRank algorithm

In order to apply the PR algorithm to the Diamond League event, we constructed a [33×33] adjacency matrix for all the competing athletes, which we updated after every race. For each race, any athlete who beat another received a ‘vote’ of 1 from the beaten athlete. So in a race comprising eight athletes, the winner would receive seven ‘votes’ from the other athletes, while the athletes who came second and third would receive six and five ‘votes’ respectively. This pattern would continue to the second to last athlete, who would receive one ‘vote’ from the last athlete, who would of course receive no ‘votes’. Where two athletes tied for a position, they were each considered to have voted for each other. After every race, the adjacency matrix was updated with the new ‘votes’ added to the existing ‘votes’ from the previous races. As such, the adjacency matrix maintained a precise updated summary of ‘who beat who’.

The updated adjacency matrices were analysed using an ‘in-house’ PR algorithm, which was used to generate vectors containing the PR scores, together with network graphs showing the connectivity between the athletes. In keeping with previous work [23], when computing the PR scores, we set the damping factor, α, to 0.85.

### D. User preference algorithm

Internet based retailers frequently rely on a ‘five star’ rating system to assess user preferences. This performs two functions; firstly it enables users to rate particular products; and secondly it allows similar products to be ranked so that retailers can compare their ‘performance’. This can be done by compiling a skew-symmetric weighted adjacency matrix from UP data, as described by Langville and Meyer [18]. Once this matrix is defined, the product ratings can easily be assessed using:

$$b=\frac{Ke}{n}$$ (9)

where, *n* is the number of products being assessed; *b* is a [n×1] vector containing the UP ranks for the various products; *K* is a [n×n] skew-symmetric adjacency matrix; and *e* is a [n×1] vector populated entirely with ones.

In order to explain the methodology associated with a typical user preference rating system, we consider the example below, in which six users (*u*_*1*_ *… u*_*6*_) rate four films (*f*_*1*_ *… f*_*4*_) to produce a UP matrix, *U*, in which the rows denote the users and the columns denote the films. From this we can see that user *U*_*5*_, for example, has given film *f*_*1*_ a rating of five stars (the highest rating score) and a rating of three stars to film *f*_*4*_.

$$U=\left[\begin{array}{cccc} 4 & 2 & & 3\\ 1 & & 3 & 2\\ & 5 & & 2\\ 4 & 5 & 1 & \\ 5 & & & 3\\ & & 2 & 2 \end{array}\right]$$ (10)

**Fig 2:**
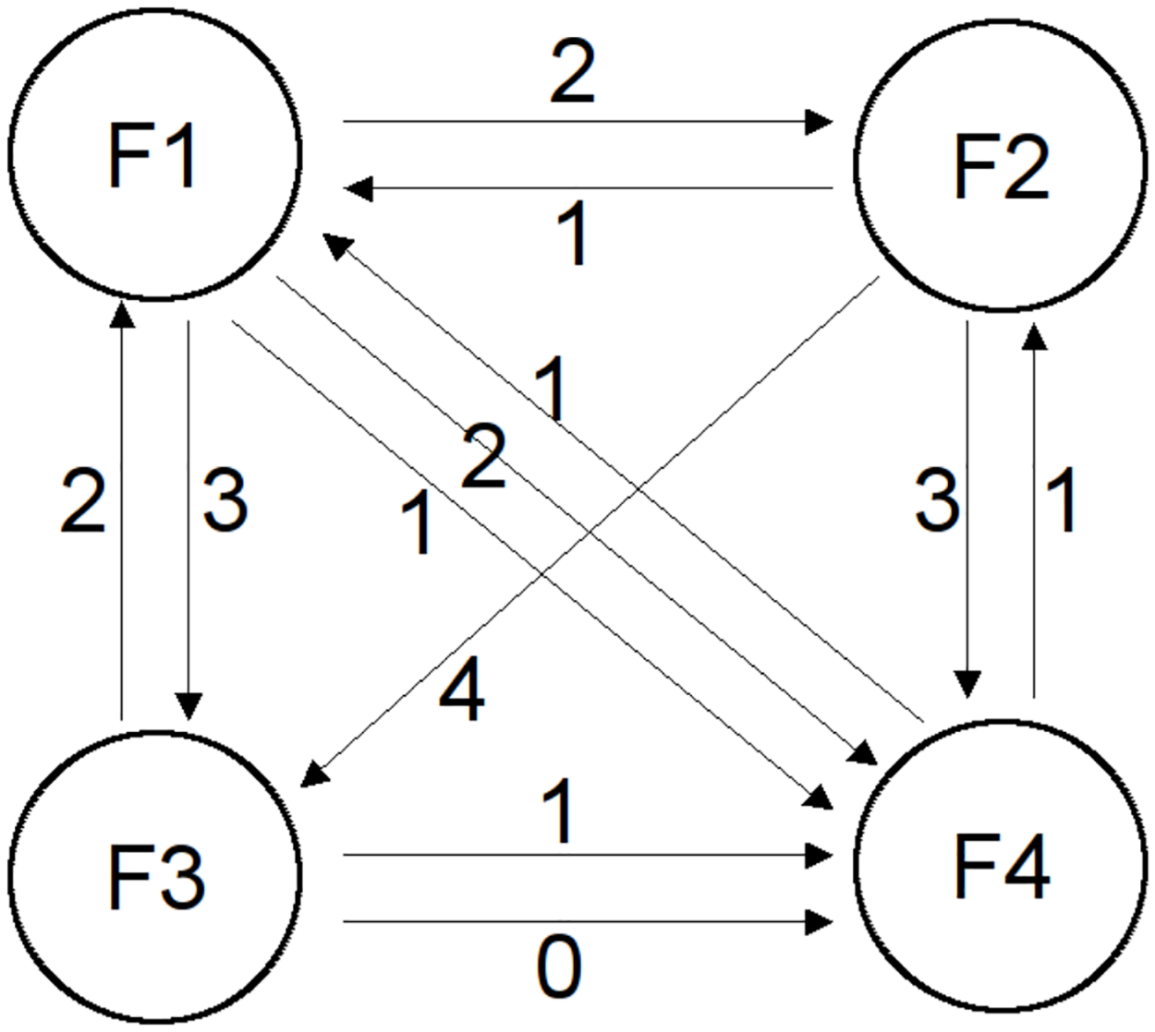
Graph for user preference rating example. The nodes represent the films and the edges represent the user ratings, with edge scores representing the numerical difference between the user ratings for the two nodes.

The graph associated with matrix, *U*, is shown in Fig 2. From this the paired [n×n] matrix *K* can be derived, with the numerical values of *K* representing the average of the score differences between the respective films and the signs representing the direction of these differences.

$$K=\left[\begin{array}{cccc} 0 & \frac{1}{2} & \frac{1}{2} & \frac{2}{3}\\ -\frac{1}{2} & 0 & \frac{4}{1} & \frac{2}{2}\\ -\frac{1}{2} & -\frac{4}{1} & 0 & \frac{1}{2}\\ -\frac{2}{3} & -\frac{2}{2} & -\frac{1}{2} & 0 \end{array}\right]$$ (11)

Once matrix, *K*, is formulated then the rating vector, *b*, can be derived using Eq (9), as follows:

$$b=\frac{Ke}{4}=\left[\begin{array}{c} 0.4167\\ 1.1250\\ -1.0000\\ -0.5417 \end{array}\right]$$ (12)

From b it can be seen that film *f*_*2*_ is ranked first, followed by *f*_*1*_, *f*_*4*_, *and lastly f*_*3*_.

### E. Application of the user preference algorithm

In order to apply the UP algorithm to the Diamond League event, we constructed a [10×33] UP matrix in which the races represented the individual ‘user evaluations’. Because the races contained varying numbers of contestants (i.e. some races comprised nine runners, while others had only seven), we decided to use a reverse scoring system, in which the athletes who came first, second and third received one, two and three ‘stars’ respectively, with the number of ‘stars’ incrementally increasing for the lower finishing places. In this way, the number of athletes competing in any given race had minimal impact on the overall rankings, because the winner would always received one ‘star’, with the second athlete always receiving two ‘stars’, and so on. The rankings were derived for the individual athletes using the methodology described above. Because we used a reverse scoring system, the final ranking vector, b, was multiplied by –1 in order to make the ranking scores positive for the leading athletes.

### F. Keener algorithm

In 1993, James Keener proposed a ranking method that utilized a non-negative matrix constructed using the results of games between competing teams [21]. Although metrics such as win ratio can be used to construct Keener's matrix, *X*, it is generally more common to use the number of points or goals that team *i* scored against team *j* [18]. Indeed, Keener suggested using a score ratio that satisfies Laplace’s rule of succession as follows:

$$a_{ij}=\frac{S_{ij}+1}{S_{ij}+S_{ji}+2}$$ (13)

where, a_*ij*_ is the value of the statistic produced when team *i* competes against team *j*; *S*_*ij*_ is the number of points team *i* scores against team *j*; and *S*_*ji*_ is the number of points team *j* scores against team *i*.

Since the Keener method utilises game scores to compute ratings, it can be susceptible to bias when teams accumulate high scores. In order to compensate for this Keener suggested applying the non-linear skewing function:

$$h(x)=0.5+0.5\left(\text{sgn}(x-0.5)\times \sqrt{\left|2x-1\right|}\right)$$ (14)

to each computed value, *a*_*ij*_, to generate a non-negative matrix that is irreducible provided that enough games have been played between the teams. By exploiting the Perron-Frobenius theorem [24] and using Eq (15), Keener was able to compute the Perron vector, (i.e. a unique vector derived from the eigenvector corresponding to the largest eigenvalue of the Keener matrix), which contains the positive ratings of all the teams involved in any given competition [18].

$$Xr_{k}=\lambda r_{k}$$ (15)

where, *X* is the Keener matrix; *r*_*k*_ is the Perron rating vector; and *λ* is the proportionality constant.

In order to explain the methodology associated with the Keener algorithm, let us consider a hypothetical mini-league in which six soccer teams (Arsenal, Chelsea, Liverpool, Stoke, Swansea and Tottenham) compete. If we assume that each team has played three matches (results shown in Table 2), then the following ‘goals scored’ adjacency matrix, *L*, can be constructed in which the rows denote the goals scored by the respective teams and the columns denote goals conceded.

**Table 2:** The results of matches in the mini- soccer league.

Match	Score
Arsenal v Swansea	2–0
Chelsea v Stoke	5–1
Liverpool v Tottenham	1–0
Swansea v Tottenham	1–4
Chelsea v Liverpool	2–1
Stoke v Arsenal	0–1
Liverpool v Chelsea	3–1
Arsenal v Tottenham	2–3
Stoke v Swansea	1–0

$$L=\left[\begin{array}{cccccc} 0 & 0 & 0 & 1 & 2 & 2\\ 0 & 0 & 3 & 5 & 0 & 0\\ 0 & 4 & 0 & 0 & 0 & 1\\ 0 & 1 & 0 & 0 & 1 & 0\\ 0 & 0 & 0 & 0 & 0 & 1\\ 3 & 0 & 0 & 0 & 4 & 0 \end{array}\right]$$ (16)

By applying Eqs (13) and (14) to the elements in matrix, *L*, and adding a small perturbation to ensure irreducibility, it is possible to compute the Keener matrix, *X*.

$$X=\left[\begin{array}{cccccc} 0.000 & 0.000 & 0.000 & 0.789 & 0.854 & 0.311\\ 0.000 & 0.000 & 0.333 & 0.854 & 0.000 & 0.000\\ 0.000 & 0.667 & 0.000 & 0.000 & 0.000 & 0.789\\ 0.211 & 0.147 & 0.000 & 0.000 & 0.789 & 0.000\\ 0.147 & 0.000 & 0.000 & 0.211 & 0.000 & 0.173\\ 0.689 & 0.000 & 0.211 & 0.000 & 0.827 & 0.000 \end{array}\right]$$ (17)

Finally, by solving Eq (15), the Perron rating vector, *r*_*k*_, can be computed, which indicates that the algorithm ranks the teams in the following order: Liverpool, Tottenham, Arsenal, Chelsea, Stoke and Swansea.

$$r_{k}=\left[\begin{array}{c} 0.195\\ 0.153\\ 0.242\\ 0.110\\ 0.079\\ 0.220 \end{array}\right]$$ (18)

### G. Application of the Keener algorithm

In order to apply the Keener algorithm to the Diamond League event, we constructed a [33×33] adjacency matrix for all the competing athletes, which we populated with data from all ten races. In order to ensure consistency with the other ranking techniques used, we adopted a ‘who beat who’ strategy, in which each athlete who beat another athlete receiving a ‘vote’ of 1 from the defeated athlete, in the similar manner to the PR algorithm. In the event of a tie between two athletes, a ‘vote’ of 0.5 was awarded to each athlete [18]. Eq (13) was then used to compute the a_*ij*_ statistic, which in this case represented the adjusted win ratio, with s_*ij*_ being the number of times athlete *i* beat athlete *j*, and s_*ji*_ being the number of occasions on which athlete *j* beat athlete *i*. For the Diamond League application, the win ratio was used rather than the score ratio, because we felt that this best reflected the ‘who beat who’ approach used in the PR algorithm.

### H. Colley algorithm

Unlike the Keener method, which takes into account the outcome of individual matches between teams, the Colley algorithm utilises only the total number of wins, losses and games played to rank competing teams. It was developed by Wesley Colley in 2002 to rate teams in match-orientated sports [19] and utilizes Laplace’s rule of succession [18] to produce a [n ×1] vector, *v*, (where, n is the number of competing teams) using Eq (19):

$$v_{i}=1+0.5(w_{i}-l_{i})$$ (19)

where, *v*_*i*_ is the combined ‘score’ of the *i*^*th*^ team; *w*_*i*_ is the number of wins of the *i*^*th*^ team; and *l*_*i*_ is the number of losses of the *i*^*th*^ team.

Colley’s method solves the linear system:

$$Cr_{c}=v$$ (20)

where, *r*_*c*_ is the Colley rating vector, which defines the ranking of the teams; and *C* is the Colley coefficient matrix defined as [13]:

C=ij{2+piifi=j,−pijifi≠j, (21)

where, *p*_*i*_ is the total number of times team *i* has played; and *p*_*ij*_ is the number of matches played between teams *i* and *j*.

If the Colley algorithm is applied to the hypothetical mini-soccer league outlined above (see Table 2), then the Colley matrix, C, is:

$$C=\left[\begin{array}{cccccc} 5 & 0 & 0 & -1 & -1 & -1\\ 0 & 5 & -2 & -1 & 0 & 0\\ 0 & -2 & 5 & 0 & 0 & -1\\ -1 & -1 & 0 & 5 & -1 & 0\\ -1 & 0 & 0 & -1 & 5 & -1\\ -1 & 0 & -1 & 0 & -1 & 5 \end{array}\right]$$ (22)

and the vector, *v*, is:

$$v=\left[\begin{array}{c} 1.5\\ 1.5\\ 1.5\\ 0.5\\ -0.5\\ 1.5 \end{array}\right]$$ (23)

By solving Eq (20), the Colley rating vector, *r*_*c*_, can be computed, which indicates that the Colley algorithm ranks the teams in the following order: Liverpool, Chelsea, Tottenham, Arsenal, Stoke and Swansea.

$$r_{c}=\left[\begin{array}{c} 0.530\\ 0.644\\ 0.674\\ 0.374\\ 0.197\\ 0.580 \end{array}\right]$$ (24)

### I. Application of the Colley algorithm

In order to apply the Colley algorithm to the Diamond League event, we constructed a [33×33] adjacency matrix in the same manner as used with Keener algorithm. We used this to calculate for each athlete, the total number of contests won and lost against the other athletes, which we then used to compute the vector, v, using Eq (19). The Colley matrix was compiled as described above, with the number of ‘matches’ being the number of times the respective athletes ran against each other.

### J. Massey algorithm

In 1997 Kenneth Massey proposed a ranking model which used a least squares approach to solve a system of linear equations expressing the relationship between team ratings and the margin of victory [20]. Massey’s method involved constructing a [m×n] matrix, *W*, recording the outcomes of m matches between n teams. Matrix, *W*, is populated according to the following rules [13], where *w*_*ki*_ is an indicator variable for the outcome of the *k*^*th*^ game for team *T*_*i*_.

W=ki{1ifteam  Ti won the kth game,−1ifteam  Ti lost the kth game,0otherwise (25)

Massey used matrix, *W*, and a vector, *y*, containing the margins of victory, to solve the following normal equation, in which *r*_*m*_ is the vector of unknown ratings.

$$W^{T}Wr_{m}=W^{T}y$$ (26)

Conveniently, Massey was able to simplify Eq (26) to:

$$Mr_{m}=d$$ (27)

where, the Massey matrix, *M*, is:

M=ij(WTW)ij={piifi=j,−pijifi≠j, (28)

*p*_*i*_ is the total number of games played by team *i*; *p*_*ij*_ is the number of matches played between teams *i* and *j*, and *d* is the vector of cumulative points differentials.

$$d=W^{T}y$$ (29)

If Massey’s method is applied to the mini-soccer league example (see Table 2), then the Massey matrix, *M*, becomes:

$$M=\left[\begin{array}{cccccc} 3 & 0 & 0 & -1 & -1 & -1\\ 0 & 3 & -2 & -1 & 0 & 0\\ 0 & -2 & 3 & 0 & 0 & -1\\ -1 & -1 & 0 & 3 & -1 & 0\\ -1 & 0 & 0 & -1 & 3 & -1\\ -1 & 0 & -1 & 0 & -1 & 3 \end{array}\right]$$ (30)

and the vector of cumulative goal differentials, *d*, is:

$$d=\left[\begin{array}{c} 2\\ 3\\ 2\\ -4\\ -6\\ 3 \end{array}\right]$$ (31)

Because Eq (27) does not necessarily have a unique solution, Massey proposed a workaround solution [18, 20], which involved replacing the last row of the matrix, *M*, with a row of ones, and the last row of the vector, *d*, with a zero as follows, thus forcing the unique solution for *r*_*m*_ shown in Eq 33.

[300−1−1−103−2−1000−2300−1−1−103−10−100−13−1111111]×[rm1rm2rm3rm4rm5rm6]=[232−4−60] (32)

$$r_{m}=\left[\begin{array}{c} -0.500\\ 1.857\\ 2.143\\ -1.714\\ -2.500\\ 0.714 \end{array}\right]$$ (33)

From vector, *r*_*m*_, it can be seen that the Massey methods ranks the teams in exactly the same order as the Colley algorithm, namely: Liverpool, Chelsea, Tottenham, Arsenal, Stoke and Swansea.

### K. Application of the Massey algorithm

In order to apply the Massey algorithm to the Diamond League event, we constructed a [33×33] adjacency matrix for all the competing athletes, which we populated with data from all ten races. Since the Massey method was developed for competitions in which teams play paired matches, we adopted a strategy that mimicked the scoring in a soccer match. So in a race comprising eight athletes, the winner was deemed to have scored seven ‘goals’ more than the last athlete, who was deemed to have scored no ‘goals’. Likewise, second and third athletes in the race were deemed to have scored six and five ‘goals’ respectively. This pattern continued until the second to last athlete, who scored only one ‘goal’ more than the last athlete. We used this matrix to calculate the cumulative points differential score for each athlete, which we compiled into vector, *p*. The Massey matrix was compiled as described above, with the number of ‘matches’ being the number of times the respective athletes ran against each other.

### L. Statistical analysis

The final rankings produced by the various algorithms were compared with each other and also with the standings computed using the official IAAF Diamond League points system. In the 2015 season, for all the races, except the final meeting in Bruxelles, four points were awarded for first place, two points for second place, and one for third place [25]. In the final race in Bruxelles the points awarded were double those awarded for the other races. However, in 2016 the IAAF changed its points system to accommodate lesser positions. Under the ‘2016’ points system, ten points were awarded for a win, six for second, four for third, three for fourth, two for fifth, and one for six [26]. As in the ‘2015’ points system double points were awarded for the final race. Because both IAAF points systems reflected different attributes, we used both methods to compute alternative final standings for the athletes, which we than compared with the standings produced using the various algorithms described above.

In order to compare the standings produced by the various ranking systems, we calculated the Kendall’s tau rank distances between the respective standings for the top 23 athletes (i.e. the number of athletes ranked by the Diamond League ‘2016’ points system), which we then normalized (Eq (34)) to compute the percentage of pairs that differed in order between the ranking lists. Kendall’s tau distance is a metric that counts the number of pair order disagreements between two ranking lists. It can be normalized to yield the fraction of discordant pairs as follows:

$$\tau_{dist}=\frac{N_{d}}{N(N-1)/2}$$ (34)

where, *τ*_*dist*_ is the normalized Kendall’s tau distance; *N* is the number of items in each ranking list; and *N*_*d*_ is the number of discordant pairs.

In addition, Pearson correlation analysis was performed using the paired ranking scores computed by the various systems for the respective athletes. Statistical analysis of the data was performed using ‘in-house’ algorithms written in ‘R’ and Matlab. For all tests, p values <0.05 were deemed to be significant.

## Results

**Fig 3:**
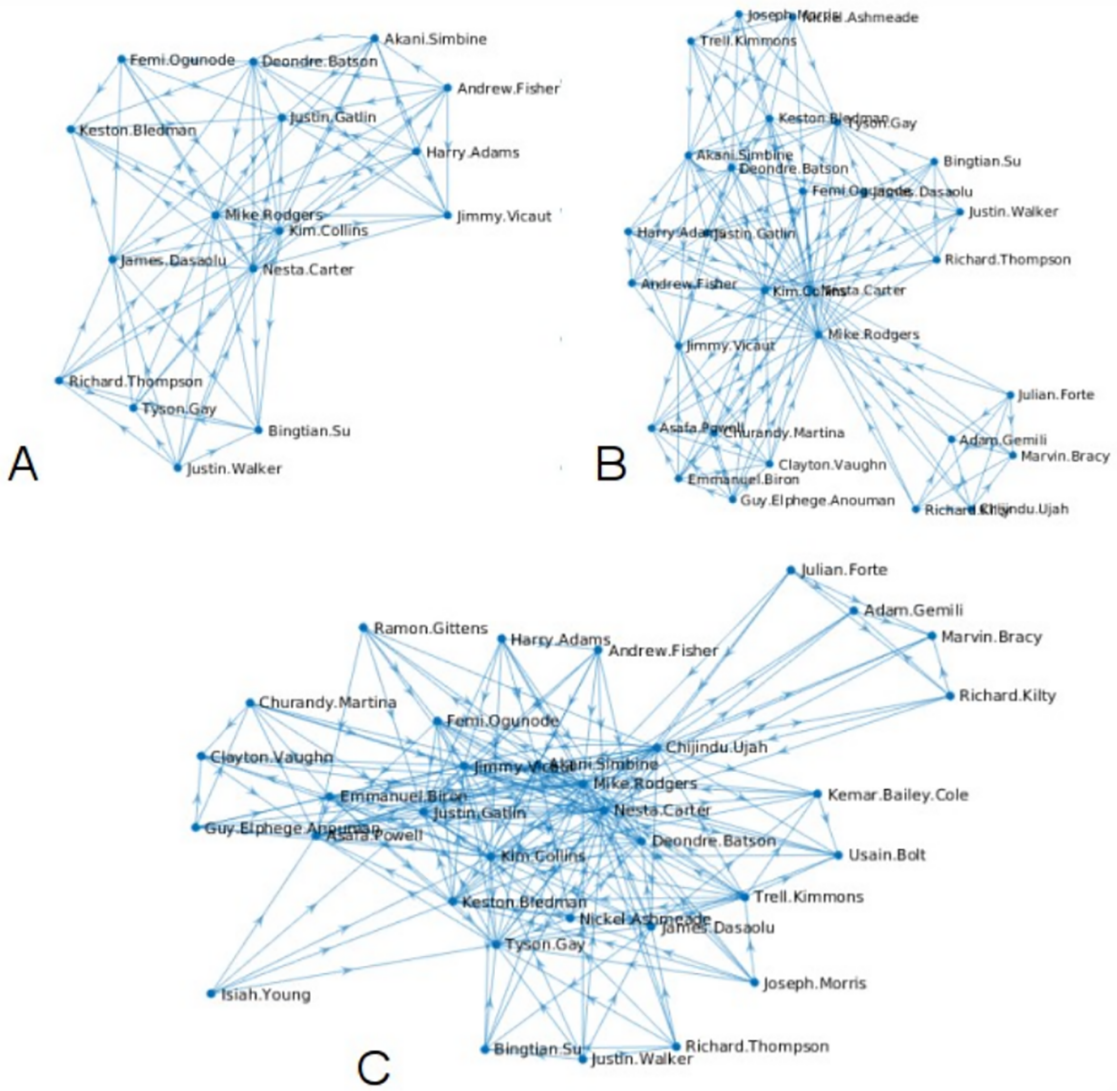
Connectivity networks between athletes after: (A) the first three races; (B) the first six races; and (C) all ten races.

The standings computed by the PR algorithm for each competing athlete after every race are presented in Table 3. From this it can be seen that as the season progressed, the number of rank scores allocated, steadily increased as more and more athletes became involved in the competition. This is illustrated in Fig 3, which shows the respective connectivity networks for the system after three, six and ten races. In these network graphs the graph edges (i.e. the straight lines) link competitors who raced against each other, with the direction of the arrow indicating who beat who. It can be seen from this that after three races (Fig 3A) the network is still relatively simple, with only 16 competitors involved in the competition. However, when all 33 athletes have become involved, after ten races, the network becomes much more complex and difficult to understand (Fig 3C).

**Table 3:** Rank scores allocated to each competing athlete after every race using the PageRank algorithm.

		Doha	Eugene	Rome	Birmingham	New York	Paris	Lausanne	Monaco	London	Bruxelles
		15^th^ May	30^th^ May	4^th^ June	7^th^ June	13^th^ June	4^th^ July	9^th^ July	17^th^ July	24^th^ July	11^th^ Sept
Athlete ID	Name	Rank	Rank	Rank	Rank	Rank	Rank	Rank	Rank	Rank	Rank
1	Harry Adams	na	na	=15	=19	=21	=25	=25	=25	=27	=28
2	Nickel Ashmeade	na	na	na	na	16	18	18	16	20	20
3	Guy-Elphege Anouman	na	na	na	na	na	=25	=25	=25	=27	=28
4	Kemar Bailey-Cole	na	na	na	na	na	na	na	na	13	14
5	Deondre Batson	7	11	9	11	10	11	11	11	14	16
6	Emmanuel Biron	na	na	na	na	na	24	24	24	26	27
7	Keston Bledman	3	5	7	9	6	9	9	9	10	11
8	Usain Bolt	na	na	na	na	na	na	na	na	8	10
9	Marvin Bracy	na	na	na	3	4	4	6	5	5	6
10	Nesta Carter	6	7	6	5	5	5	5	6	6	7
11	Kim Collins	4	4	5	7	8	8	7	8	9	9
12	James Dasaolu	8	9	12	15	15	17	17	19	22	21
13	Andrew Fisher	na	na	14	18	20	23	23	23	25	26
14	Julian Forte	na	na	na	=19	=21	=25	=25	=25	=27	=28
15	Justin Gatlin	1	1	1	1	1	2	1	1	1	1
16	Tyson Gay	na	2	3	4	3	3	4	2	3	3
17	Adam Gemili	na	na	na	8	9	10	10	10	12	13
18	Ramon Gittens	na	na	na	na	na	na	na	na	na	23
19	Richard Kilty	na	na	na	14	14	16	16	18	19	22
20	Trell Kimmons	na	na	na	na	19	22	22	22	17	18
21	Churandy Martina	na	na	na	na	na	19	19	20	23	24
22	Joseph Morris	na	na	na	na	=21	=25	=25	=25	=27	=28
23	Femi Ogunode	5	8	10	12	13	15	15	15	21	8
24	Asafa Powell	na	na	na	na	na	6	2	4	4	5
25	Mike Rodgers	2	3	2	2	2	1	3	3	2	2
26	Akani Simbine	na	na	11	13	11	12	12	12	15	15
27	Bingtian Su	na	6	8	10	12	13	13	13	16	17
28	Richard Thompson	na	10	13	17	18	21	21	21	24	25
29	Chijindu Ujah	na	na	na	16	17	20	20	14	11	12
30	Clayton Vaughn	na	na	na	na	na	14	14	17	18	19
31	Jimmy Vicaut	na	na	4	6	7	7	8	7	7	4
32	Justin Walker	na	12	=15	=19	=21	=25	=25	=25	=27	=28
33	Isiah Young	na	na	na	na	na	na	=25	=25	=27	=28

The results in Table 3 reveal that, with just one exception, the PR algorithm consistently ranked Justin Gatlin as number one throughout the season, while Tyson Gay and Mike Rogers broadly competed for second and third place. When athletes of higher quality, such as Marvin Bracy and Usain Bolt, entered the competition, they scored relatively highly, depressing the rank scores of less able competitors. Of the lower ranking athletes, Harry Adams, Guy-Elphege Anouman, Julian Forte, Joseph Morris, Justin Walker and Isiah Young only ran once and all finished last in the single race in which they were entered. As such, they all received the same low PR and were ranked in equal last place.

**Fig 4:**
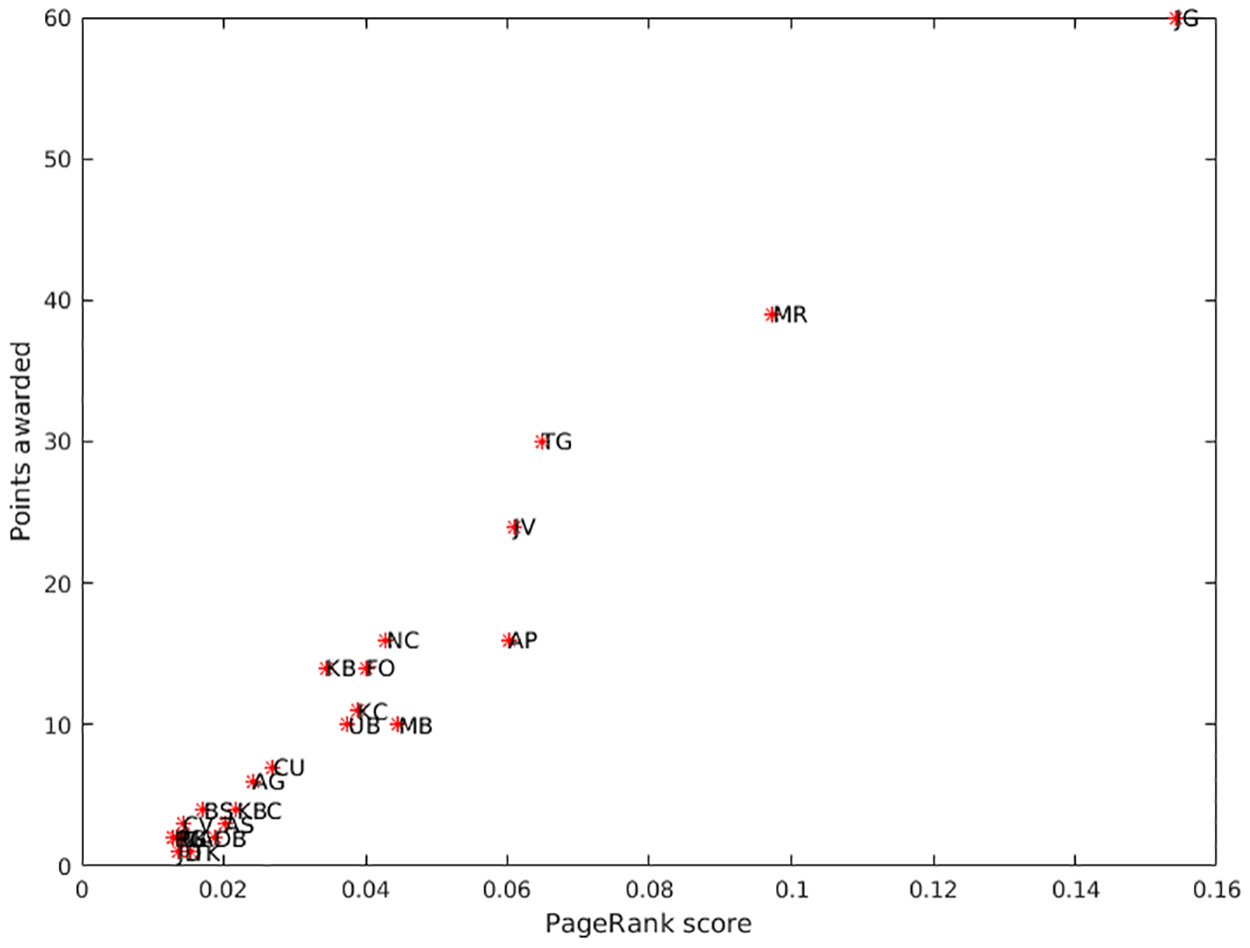
Scatter plot of the paired PageRank scores and points (calculated using the 2016 points system) awarded to the top 23 athletes.

**Fig 5:**
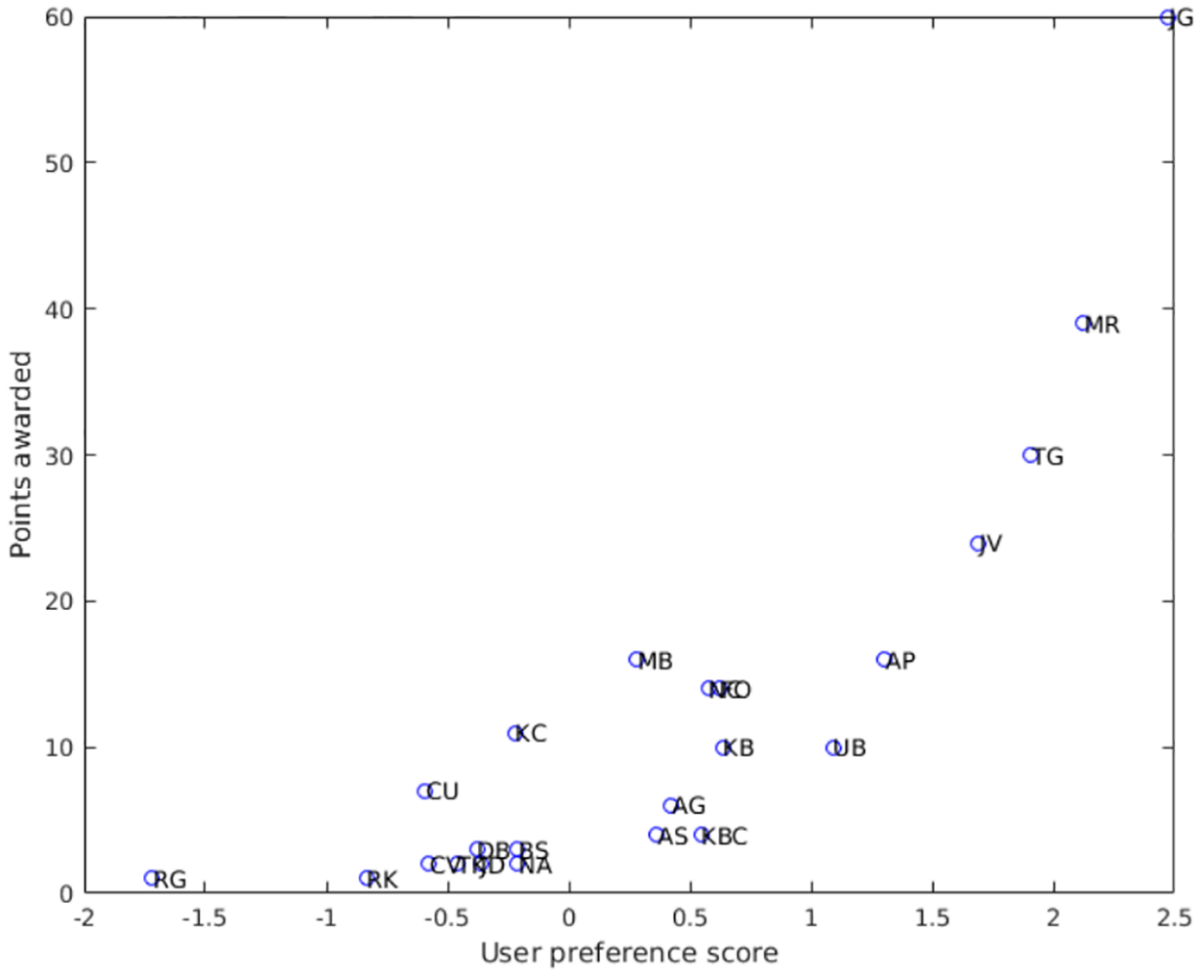
Scatter plot of the paired user preference scores and points (calculated using the 2016 points system) awarded to the top 23 athletes.

Table 4 presents the PR, UP, Keener, Colley and Massey standings for the athletes after ten races, together with fastest times achieved and the rankings calculated using the official IAAF Diamond League 2015 and 2016 points systems. This reveals that the PR standings bear a close resemblance to the rankings achieved using the Diamond League points system (particularly the 2016 system) and also, to a lesser extent, those produced by the Keener and UP algorithms. Indeed, the top five places for the PR, UP, Keener and 2016 points system standings were all identical. The Kendall’s tau distances and Pearson correlations between the various standings are presented in Table 5. From these it can be seen that the tau distance between the PR standings and ‘2016’ points system standings was 15, indicating that only 5.9% of pairs differed in their order between the two lists. The equivalent tau distances for the Keener and UP standings were 76 (30.0% pair order disagreement) and 95 (37.5% pair order disagreement) respectively, indicating that their agreement with the 2016 Diamond League points system was much less strong than that produced using the PR algorithm. The close relationship between PR scores and the ‘2016’ points is highlighted in the scatter plot shown in Fig 4. From this it can be seen that there is a very strong positive correlation (r = 0.984, p<0.001) between the two. By comparison, the ‘2016’ and UP standings displayed a less strong relationship (r = 0.845, p<0.001) (Fig 5), as did the ‘2016’ and Keener standings (r = 0.924, p<0.001). These differences are reflected in the tau distances between the PR and the UP and Keener standings, which were 87 (34.4% pair order disagreement) and 67 (26.5% pair order disagreement) respectively.

**Table 4:** Final standings computed using the various ranking algorithms together with the standings computed using methods adopted by the Diamond League.

	PageRank	User Preference	Keener	Colley	Massey	Diamond League	Diamond League	Diamond League Official
	Algorithm	Algorithm	Algorithm	Algorithm	Algorithm	2015 Points System	2016 Points System	Fastest Times
Rank Score	Rank Order (Score)	Rank Order (Score)	Rank Order (Score)	Rank Order (Score)	Rank Order (Score)	Rank Order (Points)	Rank Order (Points)	Rank Order [Time (s)]
1	J Gatlin (0.154)	J Gatlin (2.477)	J Gatlin (0.088)	J Gatlin (1.040)	U Bolt (3.955)	J Gatlin (24)	J Gatlin (60)	J Gatlin [9.74]
2	M Rodgers (0.097)	M Rodgers (2.124)	M Rodgers (0.072)	M Bracy (0.912)	J Gatlin (3.927)	T Gay (11)	M Rodgers (39)	A Powell [9.81]
3	T Gay (0.065)	T Gay (1.909)	T Gay (0.071)	U Bolt (0.907)	M Bracy (3.641)	M Rodgers (9)	T Gay (30)	J Vicaut [9.86]
4	J Vicaut (0.061)	J Vicaut (1.692)	J Vicaut (0.063)	T Gay (0.877)	A Gemili (2.641)	J Vicaut (7)	J Vicaut (24)	U Bolt [9.87]
5	A Powell (0.060)	A Powell (1.303)	A Powell (0.050)	A Powell (0.814)	A Powell (2.473)	A Powell (6)	A Powell (16)	T Gay [9.88]
6	M Bracy (0.045)	U Bolt (1.091)	N Carter (0.050)	A Gemili (0.801)	T Gay (2.403)	M Bracy (4)	N Carter (16)	M Rodgers [9.90]
7	N Carter (0.043)	M Bracy (0.636)	K Bledman (0.048)	M Rodgers (0.776)	J Vicaut (2.307)	F Ogunode (4)	F Ogunode (14)	K Bailey-Cole [9.92]
8	F Ogunode (0.040)	K Bledman (0.626)	K Collins (0.045)	J Vicaut (0.750)	K Bailey-Cole (1.954)	U Bolt (4)	K Bledman (14)	M Bracy [9.93]
9	K Collins (0.039)	F Ogunode (0.576)	U Bolt (0.043)	F Ogunode (0.744)	M Rodgers (1.849)	K Bledman (3)	K Collins (11)	C Ujah [9.96]
10	U Bolt (0.038)	K Bailey-Cole (0.546)	F Ogunode (0.042)	K Bailey-Cole (0.726)	F Ogunode (1.775)	A Gemili (2)	U Bolt (10)	A Gemili [9.97]
11	K Bledman (0.034)	A Gemili (0.424)	C Ujah (0.040)	K Bledman (0.671)	K Bledman (0.757)	N Carter (1)	M Bracy (10)	F Ogunode [9.98]
12	C Ujah (0.027)	B Su (0.364)	K Bailey-Cole (0.034)	B Su (0.647)	B Su (0.535)	K Bailey-Cole (1)	C Ujah (7)	B Su [9.99]
13	A Gemili (0.024)	N Carter (0.283)	A Simbine (0.033)	N Carter (0.534)	R Gittens (-0.188)	B Su (1)	A Gemili (6)	K Collins [9.99]
14	K Bailey-Cole (0.022)	C Vaughn (-0.212)	D Batson (0.026)	K Collins (0.505)	R Kilty (-0.359)		K Bailey-Cole (4)	N Carter [10.00]
15	A Simbine (0.020)	R Kilty (-0.212)	M Bracy (0.025)	R Gittens (0.502)	N Carter (-0.451)		B Su (4)	K Bledman [10.01]
16	D Batson (0.019)	K Collins (-0.222)	A Gemili (0.023)	C Vaughn (0.477)	C Ujah (-0.475)		A Simbine (3)	R Kilty [10.05]
17	B Su (0.017)	R Gittens (-0.364)	T Kimmons (0.022)	C Ujah (0.472)	C Vaughn (-0.835)		C Vaughn (3)	J Forte [10.06]
18	T Kimmons (0.015)	A Simbine (-0.379)	B Su (0.021)	R Kilty (0.468)	K Collins (-0.836)		D Batson (2)	T Kimmons [10.07]
19	C Vaughn (0.014)	D Batson (-0.460)	J Dasaolu (0.020)	A Simbine (0.426)	A Simbine (-1.171)		N Ashmeade (2)	A Simbine [10.08]
20	N Ashmeade (0.014)	C Martina (-0.546)	E Biron (0.020)	I Young (0.398)	D Batson (-1.835)		R Kilty (2)	D Batson [10.08]
21	J Dasaolu (0.014)	N Ashmeade (-0.576)	R Gittens (0.020)	D Batson (0.365)	I Young (-2.089)		R Gittens (2)	C Vaughn [10.08]
22	R Kilty (0.013)	C Ujah (-0.591)	N Ashmeade (0.019)	T Kimmons (0.350)	N Ashmeade (-2.111)		T Kimmons (1)	I Young [10.11]
23	R Gittens (0.013)	R Thompson (-0.606)	C Vaughn (0.015)	N Ashmeade (0.324)	T Kimmons (-2.280)		J Dasaolu (1)	N Ashmeade [10.11]
24	C Martina (0.013)	I Young (-0.636)	A Fisher (0.014)	C Martina (0.323)	J Forte (-2.359)			R Gittens [10.11]
25	R Thompson (0.012)	J Forte (-0.636)	C Martina (0.014)	A Fisher (0.256)	C Martina (-2.626)			C Martina [10.12]
26	A Fisher (0.012)	T Kimmons (-0.833)	H Adams (0.013)	R Thompson (0.247)	E Biron (-3.122)			J Dasaolu [10.12]
27	E Biron (0.012)	J Walker (-0.849)	R Kilty (0.012)	J Forte (0.245)	J Dasaolu (-3.289)			A Fisher [10.14]
28	J Walker* (0.011)	J Morris (-0.849)	GE Anouman (0.011)	J Dasaolu (0.234)	R Thompson (-3.465)			E Biron [10.17]
29	H Adams* (0.011)	A Fisher (-0.849)	R Thompson (0.010)	E Biron (0.217)	A Fisher (-3.601)			H Adams [10.24]
30	J Forte* (0.011)	GE Anouman (-1.091)	I Young (0.010)	H Adams (0.165)	GE Anouman (-4.391)			R Thompson [10.27]
31	J Morris* (0.011)	H Adams (-1.121)	J Walker (0.010)	GE Anouman (0.159)	J Walker (-4.465)			J Walker [10.28]
32	GE Anouman* (0.011)	E Biron (-1.303)	J Morris (0.009)	J Walker (0.147)	H Adams (-4.601)			GE Anouman [10.32]
33	I Young* (0.011)	J Dasaolu (-1.717)	J Forte (0.007)	J Morris (0.116)	J Morris (-4.670)			J Morris [10.45]

* Athletes finished last in their respective races and are therefore ranked in race order.

**Table 5:** Kendall’s tau distance and percentage disagreement in pair order, together with Pearson correlation r-values, between the top 23 athletes in each of the respective standings.

	PageRank Tau distance (% disagreement) [r value]	User Preference Tau distance (% disagreement) [r value]	KeenerTau distance (% disagreement) [r value]	Colley Tau distance (% disagreement) [r value]	Massey Tau distance (% disagreement) [r value]	Diamond League 2015 Tau distance (% disagreement) [r value]
PageRank						
User Preference	87 (34.4%) [0.851***]					
Keener	67 (26.5%) [0.901***]	102 (40.3%) [0.863***]				
Colley	99 (39.1%) [0.732***]	48 (19.0%) [0.909***]	108 (42.7%) [0.770***]			
Massey	110 (43.5%) [0.674***]	59 (23.3%) [0.877***]	109 (43.1%) [0.727***]	19 (7.5%) [0.989***]		
Diamond League 2015	na (na) [0.953***]	na (na) [0.876***]	na (na) [0.831***]	na (na) [0.718**]	na (na) [0.516]	
Diamond League 2016	15 (5.9%) [0.984***]	95 (37.5%) [0.845***]	76 (30.0%) [0.924***]	102 (40.3%) [0.681***]	114 (45.1%) [0.594**]	na (na) [0.944***]

** Pearson r value significant at p<0.01

*** Pearson r value significant at p<0.001

While the Colley and Massey algorithms yielded standings that were very similar to each other (tau distance = 19, 7.5% pair order disagreement), the standings produced by both these methods were markedly different from those produce by either the PR and Keener algorithms, or indeed the Diamond League points systems. For example, the Colley and Massey algorithms ranked Usain Bolt and Marvin Bracy much higher, and Mike Rogers much lower, than any of the other methods. Notwithstanding this, some similarities were observed between the UP standings and the Colley and Massey standings, with the tau distances between these ranking lists being only 48 (19.0% pair order disagreement) and 59 (23.3% pair order disagreement) respectively, much smaller than the equivalent distances between the UP standings and the PR and Keener algorithms.

## Discussion

The results of our study are promising and suggest that graph based algorithms have considerable potential as tools for ranking the performance of track athletes, without the need for complex tables. Currently, the ‘All-athletics’ World Rankings [27, 28] rely on a complicated system in which athletes accumulate points as they compete in IAAF approved competitions. For any given competition the overall performance score of the athlete is the sum of the ‘result score’ (based on the time achieved) and a ‘placing score’ (generally based on the athletes placing in the final of the competition) [27]. The results score is awarded for the result (i.e. time) achieved according to the IAAF Scoring Tables of Athletics [3] and is modified depending on factors such as wind speed and hand timing [27]. By comparison, placing scores are awarded only to those who reach the final of a competition (or semi-final in the case of the Olympic Games and the IAAF World Outdoor Championships), with competitions allocated different amounts of points depending on their perceived quality of the event. For example, while winning a track event at a IAAF Diamond League meeting will earn a placing score of 170, winning the same event at a IAAF World Challenge meeting will only earn 100 points [28]. As such, the IAAF system does not primarily assess ‘who beat who’, but rather, relies more on the times achieved and a series arbitrary classifications designed to reflect the quality of the competition. Consequently, there is the potential for subjectivity to enter the ranking system. By comparison systems such as the PR and UP algorithms are more objective and are much simpler, relying purely on a ‘who beat who’ matrix, no matter the level of competition, thus eliminating the need for complex tables. Furthermore, because the IAAF ranking system only allocates placing scores to the top athletes who reach the track finals, it does not reflect the race competitiveness (i.e. placings) of the lesser athletes, something that can be problematic for coaches wanting to assess the true performance of developing athletes. Thus the development of graph based algorithms capable of objectively ranking athletes, particularly developing athletes, may be of considerable benefit to all those involved in athletics.

In the present study we used the 2015 Diamond League 100m races because collectively they represented an asymmetric system of manageable size, which could easily be assessed. The Diamond League also had the added bonus of a points system, which we could use for comparison purposes. While the Diamond League points system reflects the idiosyncrasies of the competition, and therefore should not be considered definitive, it nonetheless proved a useful tool with which to assess the performance of the ranking algorithms. From Table 4, it is clear that the PR algorithm yielded very similar results to those achieved using the ‘2016’ Diamond League points system. This was particularly true for the top ranking athletes, most of who appeared in identical or similar positions in the respective standings for both methods. However, greater variability was observed between the two sets of standings for the lower ranked competitors. This is probably due to the fact that the Diamond League points system allocates placing points, irrespective of the quality of the competitors involved. So in a weak race, a lesser athlete might score more highly than would otherwise be the case if the competition were stronger, with the result that the standings may not accurately reflect the true performance of that athlete. Also, because the Diamond League points system is integer based, this means that it tends to lose definition when the point scores are low, with the result that points scored in a weak race can make a considerable difference to the rank position amongst the weaker athletes. In comparison, because the PR system has greater definition and accurately reflects ‘who beat who’, it is more able to capture true race performance and distinguish between athletes in the lower reaches of the standings.

In our PR model an athlete who beats another athlete can be considered as receiving a ‘vote’ from the beaten athlete. Consequently, the winner of a race involving eight athletes will receive seven votes. However, in the PR model these votes are not all equal, because beaten athletes with a higher PR score will make a greater contribution to the PR score of the race winner, than those from beaten athletes exhibiting a low PR score. As such, the PR algorithm tends to favour individuals who race more often and perform reasonably well (e.g. Mike Rodgers, who raced many times and was placed second or third in most races), over outstanding athletes, such as Usain Bolt, who participate sparingly. However, the same would generally be true for a points based system. As such, both approaches tend to favour athletes who perform consistently well, over those who put in outstanding ‘one-off’ performances. So although the PR algorithm and the points system utilise very different methodologies, in the context of the Diamond League, they appear to achieve very similar results.

When compared with the other ranking systems, the PR algorithm produced results that were most similar to those of the Keener algorithm, with a 26.5% pair order disagreement between the two ranking lists. This suggests that the Keener algorithm, as executed here, produces standings that capture some of the attributes displayed in the PR standings, and indicates that it may also have potential as a tool for assessing track athletes. Having said this, it is noticeable that the Keener algorithm ranked Marvin Bracy relatively lowly, despite this athlete winning the race in which he competed. This appears to be due to the fact that only seven athletes competed in Bracy’s race, whereas the Keener algorithm ranked Usain Bolt six places above Bracy, primarily because he won a race in which nine athletes competed. As such this represents a serious limitation of the Keener method, which may impede its application to track athletics.

The points based scoring system used by the Diamond League reflects the fact that it is a league, in which consistency is rewarded over ‘one-off’ victory. So we have the paradoxical situation where Kim Collins, who entered six races and whose best position was fourth, scored more points (under the 2016 system) than Usain Bolt (multiple Olympic champion) who finished first in the only race in which he ran. Although the PR algorithm also placed Kim Collins above Usain Bolt, the UP algorithm did not, placing Usain Bolt 6^th^ and Kim Collins 16^th^, suggesting that the two algorithms detected different nuances. So while the UP, PR and 2016 points systems all agreed about the first five standings, below this the results for the UP algorithm diverged from those produced by the other two approaches. The primary reason for this is that the UP algorithm tends to average the scores achieved so that ‘one-off’ victors will tend to be ranked higher than more mediocre athletes who have competed many times. As such, the UP algorithm appears to share some of the characteristics of the Colley and Massey algorithms, which both ranked ‘one-off’ victors highly. Indeed, it is noticeable (Table 5) that there was relatively little pair order disagreement between the ranking lists produced by the Colley, Massey and UP algorithms. Consequently, these algorithms may be better suited to predicting future performance, say in knockout tournaments, rather than assessing performance in competitions such as the Diamond League. The differences between the PR and UP algorithms are starkly highlighted in Figs 4 and 5. Not only did the PR algorithm rank the athletes in a similar order to the Diamond League ‘2016’ points system, it did so using broadly similar intervals to the Diamond League method, as demonstrated by the general linearity of the scatter plot in Fig 4. By comparison, the scatter plot in Fig 5 displays marked non-linearity between the results of the UP algorithm and the Diamond League system.

Linear algebra based sports ranking models such as the Coley matrix method [13, 19] and the Massey least squares system [13, 20] have been used for many years to rate teams in the Bowl Championship Series in American college football [18]. While these algorithms are well suited to team sports where each match consists of two teams playing each other, they are more difficult to apply in sports such as track athletics, where eight athletes may compete against each other in a single race, and where athletes compete in different numbers of races. Attempts have been made to address this later point by introducing the concept of a ‘super-user’ into the competition, where unequal numbers of games have been played, with promising results [29]. However, this does not address the former point, and so when applying the Colley and Massey algorithms to the Diamond League, we had to adapt them so that the athletes mimicked ‘teams’ playing each other. While the standings produced by both these algorithms tended to rank strong ‘on-off’ athletes such as Usain Bolt and Marvin Bracy very highly, overall they did not reflect the structure and ethos of the Diamond League. By comparison, the PR algorithm appears much better suited to track athletics tournaments such as the Diamond League. Prior to the present study, the PR algorithm had been used to rate the performance of teams in the NFL [11–13], international soccer teams [14], and individual soccer players [15, 16], but not track athletes. As such, there was no prior precedence regarding the construction of the adjacency and UP matrices used in the study. Consequently, one of the major challenges associated with the study was to develop a suitable methodology for ensuring that these matrices accurately reflected the characteristics of the competition. After due consideration, for the PR algorithm, we decided to construct an adjacency matrix in which the more successful athletes received ‘votes’ from the defeated athletes whom they beat. So for a race containing eight athletes, the winner would receive seven votes, while the last athlete would receive none. This meant that at the end of the ten race series, there were a few athletes who were placed equal bottom of the standings by the PR algorithm simply because they competed only once and came last in their respective races. With regard to the UP algorithm, one major challenge was how best to cope with the varying number of athletes in each race. If, as is normal for Internet retailing, we had adopted a ‘five star’ system, then only the first five athletes in each race would have been ranked. Conversely, if we had awarded ‘stars’ to every athlete that competed, then the winner of a race containing nine competitors would receive two more ‘stars’ than an athlete who won a race involving just seven individuals. We therefore decided on the reverse ‘star’ strategy described in the methodological section above, because this best preserved scoring parity amongst the leading athletes. This strategy did however have a downside, in that an athlete who finished last in a race involving just seven athletes would receive more ‘stars’ than one involved in a race with eight athletes. As such, this led to a few anomalies amongst the lower ranked athletes for the UP algorithm. For example, the UP algorithm ranked Julian Forte relatively high (twenty-fifth) compared to other athletes, despite the fact that he finished last in his respective race, simply because that race contained just seven athletes.

The purpose of the study presented here was not to develop the perfect ranking algorithm, but rather to explore the potential for applying Internet related algorithms in a sporting context were competition is fragmented. With respect to this both the PR and UP algorithms appear to have potential. Because they are both essentially ‘who beat who’ models that automatically rank the contestants, they minimise any subjectivity that would otherwise be involved. Consequently, they have the potential to objectively rank individuals and teams in sports where competition between competitors can be sparse, such as in boxing, tennis and athletics, or in international soccer and rugby. In these sports, lower ranked competitors frequently compete at a more regional level, making comparisons between individuals and teams difficult. In athletics, the ability to assign a ‘fine tuned’ score to lesser ranked athletes based solely on who beat who, rather than an arbitrary points system, may be something of particular interest to coaches seeking to assess the true performance of developing athletes, and also sporting authorities seeking to prioritise funds. Such athletes often fail to reach the finals of competitions and therefore, under the IAAF system, are not awarded a placing score, making it difficult for the coaches to assess their true performance.

While in the present study we used race position to construct the respective adjacency and UP matrices, we are aware that the difference in race time between athletes was also an option. However, in this study we decided against this because of potential differences between race tracks and variations in weather conditions, which might have compromised our results. Consequently, both the PR and UP models are essentially ‘who beat who’ methods that take no account of race times. While this approach appears well suited to the ethos of the Diamond League, we are conscious that our models could be improved by incorporating race times and plan to investigate this in the future. We are also aware that we have ignored other ranking systems that might prove useful with regard to track athletes, the foremost of which is perhaps Elo’s system which has been used for many years to rate chess players [18], and has latterly been applied to the NFL [30], soccer [31] and even the study of animal behaviour [32]. Elo’s system uses the difference in the ratings between two players to predict the outcome of any given contest, but has an interesting self-correcting mechanism, which may have applicability to track athletics. After every contest, the winning player takes points from the loser, with the amount of rating points transferred governed by the difference between the prior ratings of the two players. For any given contest, if the high-ranked player wins, then only a few points are taken from the low-ranked player. However, if an upset occurs and the lower ranked player wins, then many rating points are transferred from the high-rank loser. Consequently, Elo’s system is self-correcting, with players whose rating is too low, in the long run doing better than their initial rating might predict, with the result that they gain points until their rating reflects their true performance level. As such, Elo’s system shares some similarities with the PR algorithm, insomuch that they both consider prior rating positions when calculating outcome ranks. However, unlike the PR algorithm, which updates the whole directed graph of the system after every contest, Elo’s method restricts itself to updating the ratings of just the players involved in any given match [32].

In conclusion, we have shown that the PR and UP algorithms, when utilised with a simple ‘who beat who’ matrix, can be used to accurately rank track athletes. With specific reference to the IAAF Diamond League men’s 100m events, the PR model produces very similar results to those achieved using the Diamond League ‘2016’ points scoring system, but with more definition between the athletes, particularly in the lower ranks. By comparison, the UF algorithm captures other characteristics and may be more suitable for predicting future performance, say in knockout tournaments. As such, the algorithms appear to have considerable potential for objectively assessing the relative performance of track athletes, without the need for complicated points equivalence tables. Importantly, because the algorithms utilise a ‘who beat who’ model, they automatically adjust for the strength of the competition, thus avoiding the need for subjective decision making.

## References

[1] Till K, Cobley S, Morley D, O'Hara J, Chapman C, Cooke C. The influence of age, playing position, anthropometry and fitness on career attainment outcomes in rugby league. Journal of sports sciences. 2015;34(13):1240–5. doi: 10.1080/02640414.2015.1105380 26512761 10.1080/02640414.2015.1105380

[2] Till K, Jones BL, Cobley S, Morley D, O'Hara J, Chapman C, et al Identifying Talent in Youth Sport: A Novel Methodology Using Higher-Dimensional Analysis. PLoS One. 2016;11(5):e0155047 doi: 10.1371/journal.pone.0155047 27224653 10.1371/journal.pone.0155047 PMC4880304

[3] Spiriev B, Spiriev A. IAAF scoring tables of athletics: International Association of Athletics Federations; 2014.

[4] RFL. Rugby Foofball League: Section B3: League competition rules2016 1st December 2016. http://media.therfl.co.uk/docs/Section%20B3%20-%20League%20Competiton%20Rules_Final%20PDF.pdf.

[5] Robinson J. Understanding the Premier League2016 1st December 2016. http://worldsoccer.about.com/od/soccer101/a/101_Prem.htm.

[6] Premiership_Rugby. League Rules2016 1st December 2016. http://rd.premiershiprugby.com/matchcentre/tables/index.php#u7BeJIr0kp3sZAey.97.

[7] OWG. Official world golf ranking: How the ranking system works2016 30th November 2016. http://www.owgr.com/about.

[8] ATP. ATP world rankings: What is the ranking structure and formula for 2016?2016 1st December 2016. http://www.atpworldtour.com/en/rankings/rankings-faq.

[9] Brin S, Page L. The anatomy of a large-scale hypertextual web search engine. Computer Networks and ISDN Systems. 1998;33:107–17.

[10] Brin S, Page L. Reprint of: The anatomy of a large-scale hypertextual web search engine. Computer Networks. 2012;56(18):3825–33.

[11] Zack L, Lamb R, Ball S. An application of Google's PageRank to NFL rankings. Involve, a Journal of Mathematics. 2012;5(4):463–71.

[12] Govan AY, Meyer CD, editors. Ranking national football league teams using google's pagerank. AA Markov Anniversary Meeting; 2006; Charleston: Boson Books.

[13] Govan AY. Ranking theory with application to popular sports. Raleigh: North Carolina State University; 2008.

[14] Lazova V, Basnarkov L. PageRank Approach to Ranking National Football Teams. arXiv preprint arXiv:150301331. 2015.

[15] Pena JL, Touchette H, editors. A network theory analysis of football strategies Sports Physics: Euromech Physics of Sports Conference 2012; Palaiseau: Proc. 2012, Editions de l'Ecole Polytechnique.

[16] Brandt M, Brefeld U. Graph-based Approaches for Analyzing Team Interaction on the Example of Soccer. Machine Learning and Data Mining for Sports Analytics; 11th September; Porto2015.

[17] Balreira EC, Miceli BK, Tegtmeyer T. An Oracle method to predict NFL games. Journal of Quantitative Analysis in Sports. 10(2):183–96.

[18] Langville AN, Meyer CD. Who's# 1?: the science of rating and ranking. Princeton: Princeton University Press; 2012.

[19] Colley WN. Colley's bias free college football ranking method: The Colley matrix explained. Princeton University, Princeton 2002.

[20] Massey K. Statistical models applied to the rating of sports teams. Bluefield College 1997.

[21] Keener JP. The Perron-Frobenius theorem and the ranking of football teams. SIAM review. 1993;35(1):80–93.

[22] 2015 Diamond League results [Internet]. 2015 [cited 4th September2016]. https://www.diamondleague.com/lists-results/archive/2015/.

[23] Bressan M, Peserico E. Choose the damping, choose the ranking? Journal of Discrete Algorithms. 2010;8(2):199–213.

[24] Meyer CD. Chapter 8: Perron-Frobenius theory of nonnegative matrices Matrix analysis and applied linear algebra. Philadelphia: Society for Industrial and Applied Mathematics; 2000.

[25] How it works: IAAF Diamond League 2015 media guide [Internet]. IAAF. 2015 [cited 4th September 2016]. https://www.diamondleague.com/fileadmin/IDL_Default/files/documents/2015/2015_IDL_media_guide.pdf.

[26] The Diamond Race Rules and Points System [Internet]. IAAF. 2016 [cited 4th September 2016]. https://www.diamondleague.com/rules/.

[27] Rules of the All-Athletics World Rankings—2016 [Internet]. All-athletics.com. 2016 [cited 4th September 2016]. http://www.all-athletics.com/en-us/rules-all-athletics-world-rankings-2016.

[28] Rules of the All-Athletics World Rankings—2016—Overall Rankings [Internet]. All-athletics.com. 2016 [cited 4th September 2016]. http://www.all-athletics.com/en-us/rules-all-athletics-world-rankings-2016-overall-rankings.

[29] Chartier TP, Harris J, Hutson KR, Langville AN, Martin D, Wessell CD. Reducing the Effects of Unequal Number of Games on Rankings. IMAGE-The Bulletin of the International Linear Algebra Society. 2014;52(1).

[30] Paine N. NFL Elo Ratings Are Back!2015 10th March 2017. https://fivethirtyeight.com/datalab/nfl-elo-ratings-are-back/.

[31] World football Elo ratings [Internet]. 1997 [cited 10th March 2017]. http://www.eloratings.net/.

[32] Albers PCH, de Vries H. Elo-rating as a tool in the sequential estimation of dominance strengths. Animal Behaviour. 2001;61:489–95.

